# Effect of afzelin on inflammation and lipogenesis in particulate matter-stimulated *C. acnes-*treated SZ95 sebocytes

**DOI:** 10.3389/fmed.2025.1518382

**Published:** 2025-01-29

**Authors:** Ji Yeon Hong, Yong Hee Choi, Yoon Jin Roh, Mi-Kyung Lee, Christos C. Zouboulis, Kui Young Park

**Affiliations:** ^1^Department of Dermatology, Chung-Ang University Hospital, Chung-Ang University College of Medicine, Seoul, Republic of Korea; ^2^Department of Laboratory Medicine, Chung-Ang University Hospital, Chung-Ang University College of Medicine, Seoul, Republic of Korea; ^3^Departments of Dermatology, Venereology, Allergology and Immunology, Städtisches Klinikum Dessau, Brandenburg Medical School Theodor Fontane and Faculty of Health Sciences Brandenburg, Dessau, Germany

**Keywords:** *C. acnes*, inflammation, afzelin, particulate matter, sebocyte, acne vulgaris

## Abstract

**Background:**

Afzelin, a flavonoid (kaempferol 3-O-α-L-rhamnopyranoside) isolated from *Thesium chinense*, is known for its potent anti-inflammatory properties. However, its effects on the molecular aspects of inflammation and lipogenesis in SZ95 sebocytes has not been investigated.

**Objective:**

This study aimed to (i) investigate inflammatory and sebum secretion changes when a *Cutibacterium acnes*-treated immortalized human sebocyte cell line (SZ95) is exposed to particulate matter (PM) and (ii) examine the effects of afzelin on these.

**Methods:**

To investigate the effect of afzelin on PM- and *C. acnes*-treated SZ95 sebocytes, we injected heat-killed *C. acnes* into SZ95 cells to induce acne-like status. Thereafter, the SZ95 sebocytes were treated with PM and subsequently with afzelin. The gene expression profile was determined using real-time polymerase chain reaction analysis, and protein expression was confirmed via western blotting and immunofluorescence. Intracellular lipid droplet formation was investigated using Nile Red O staining.

**Results:**

PM treatment upregulated the mRNA and protein expression levels of inflammatory cytokine and lipogenic genes in *C. acnes*-treated SZ95 sebocytes. Furthermore, intracellular lipid-droplet accumulation increased when *C. acnes*-stimulated SZ95 cells were exposed to PM. Interestingly, the upregulated inflammatory and lipogenic gene expression induced by *C. acnes* and PM was attenuated by afzelin treatment.

**Conclusions:**

This study's findings indicate that PM potentially aggravates acne by acting on both inflammation and sebum secretion. They also reveal afzelin's ability to suppress these phenomena by not only suppressing inflammatory cytokine expression but also inhibiting sebogenesis. These findings confirm afzelin's potential therapeutic role in improving PM-exacerbated acne.

## Introduction

Acne, a chronic inflammatory condition of the pilosebaceous unit, is globally recognized as a highly prevalent dermatological affliction (1). Contributing factors to acne pathogenesis include augmented sebum production or changes in sebum components, follicular hyperkeratinization, colonization by *Cutibacterium acnes*, inflammatory responses, and an interplay between genetic and environmental influences (2, 3).

Particulate matter (PM) is a pervasive airborne pollutant, comprising a complex mixture of solid and liquid particles dispersed throughout the air. Air pollution represents a significant public health issue in numerous cities worldwide. Among the various types of air pollutants, PM is deemed one of the most detrimental (4). The skin, as the foremost barrier, is directly exposed to environmental pollutants and is susceptible to damage and a spectrum of skin diseases owing to such exposure (5, 6). PM reportedly triggers inflammatory reactions and impairs the skin barrier, potentially exacerbating conditions such as acne, atopic dermatitis, psoriasis, and allergic responses (2, 5, 7).

The clinical impact of PM varies with its size, with smaller particles such as PM2.5 being particularly concerning. Due to their smaller size, PM2.5 particles have a larger surface area-to-mass ratio, enabling them to carry more toxic substances and penetrate deeper into the skin. This can disrupt the skin barrier, induce oxidative stress, and promote inflammation, potentially exacerbating skin conditions like acne. In contrast, larger particles, such as PM10, tend to cause more superficial irritation. Despite the growing recognition of PM's role in dermatological disorders, further research is needed to clarify the size-dependent effects of PM on skin health.

Upon contact with the skin, PM can infiltrate the stratum corneum, hair follicles, and sweat glands, thereafter entering cells and compromising mitochondria, leading to the generation of reactive oxygen species (ROS). This process potentially induces oxidative stress (8, 9). The resultant ROS may cause skin inflammation by upregulating pro-inflammatory cytokine expression (10). Furthermore, exposure to PM has been linked to increased sebum production (3) and alterations in hormonal activity (11), potentially culminating in obstructed hair follicles, inflammation, and the proliferation of *C. acnes* (12, 13).

Afzelin (3-O-α-L-rhamnopyranoside) is a flavonoid extracted from *Thesium chinense* Turcz, a plant commonly found in Korea and China. Research has indicated that afzelin exhibits anti-inflammatory, anticancer, and antibacterial activities, along with DNA-protective and antioxidant effects in ultraviolet B-exposed human skin cells (14–16). Our previous study revealed that afzelin exerts intracellular ROS-scavenging effects on PM-treated HaCaT cells and inhibits pro-inflammatory cytokine expression in these cells (17). Therefore, afzelin is expected to exhibit potential in preventing PM-induced inflammatory skin diseases, including acne vulgaris.

Furthermore, previous studies have shown that exposure to PM correlates with increased visits to the clinic among adolescent and adult patients with acne (18, 19). However, literature on the association between PM exposure and acne is still emerging, and individual studies have exclusively focused on the individual effects of either PM or *C. acnes* on sebocytes (20–23). To date, no study has explored the effects of PM on SZ95 sebocytes *in vitro* following treatment with *C. acnes*. In a prior *in vivo* study, mice pre-treated with *C. acnes* and subsequently exposed to PM displayed increased inflammatory biomarker and sebum production (24, 25).

While the exact mechanisms underlying acne are yet to be fully elucidated, various exposome factors, such as pollutants, nutrition, medication, occupational influences, and climatic conditions, may play a role in the disease's progression, severity, and treatment effectiveness. Therefore, the present study aimed to evaluate the impact of PM on *C. acnes*-treated SZ95 cells and investigate the protective effects of afzelin in a PM-exposed acne cell model.

## Materials and methods

### Materials

Standard reference materials (1649b) were purchased from the National Institute of Standards and Technology (Gaithersburg, MD, USA) and dispersed in serum-free medium. *C. acnes* was obtained from the Korean Collection for Type Cultures (Daejeon, Korea). Afzelin was acquired from ChemFaces (Wuhan, China).

For Western blot analysis, glyceraldehyde-3-phosphate dehydrogenase (GAPDH), RELA/NFκB p65, and SREBP-1 antibodies were procured from Santa Cruz Biotechnology (Santa Cruz, CA, USA). p-RELA/NFκB p65, COX-2, fatty acid synthase (FAS), IL-6, and peroxisome proliferator-activated receptor gamma (PPARγ) antibodies were secured from Cell Signaling Technology (Danvers, MA, USA), and IL-1 beta antibodies were purchased from Abcam (Cambridge, UK). Primer sequences for qRT-PCR used in this study are provided as Supplementary data.

### Cell culture and reagents

Immortalized human SZ95 sebocytes (a patented cell line; gifted by Professor Christos C. Zouboulis, Department of Dermatology, Venereology, Allergology and Immunology, Dessau Medical Center, Theodore Fontane Medical University of Brandenburg, Germany) (26) were cultured in Dulbecco's Modified Eagle Medium/Nutrient Mixture F-12 (DMEM/F-12) in a 1:1 mixture (1×) (Welgene Inc., Daegu, Korea) supplemented with 10% fetal bovine serum (FBS), 1% penicillin–streptomycin (both from Gibco, Invitrogen, Carlsbad, CA, USA), and 5 ng/mL human epidermal growth factor (Sigma, St. Louis, MO, USA) at 37°C in a humidified atmosphere with 5% CO_2_ (27). When the cultures had reached confluence, the cells were treated with 0.05% trypsin/0.53 mmol/L ethylenediaminetetraacetic acid (Welgene Inc., Daegu, Korea) for 5–10 min at 37°C. The medium was replaced every 2–3 days.

### Bacterial culture

We used *C. acnes* KCTC 3314, a standard reference strain from the Korean Collection for Type Cultures, known for its role in assessing inflammatory responses. Reinforced clostridial liquid and solid medium (Difco Laboratories, Detroit, MI, USA) was used to grow *C. acnes* for 3–4 days at 37°C under anaerobic conditions (5% H_2_, 5% CO_2_, and 90% N_2_).

Cultured *C. acnes* isolates were centrifuged at 2,000 rpm for 10 min at 4°C and subsequently washed three times with cold phosphate-buffered saline (PBS). Finally, the bacterial count was estimated by measuring the optical density of the suspension at 600 nm (OD_600_) using a spectrophotometer. As we had previously observed OD_600_ = 1.0 to be equivalent to 7.0 × 10^8^ colony forming units (CFU) per 1 mL, we adjusted the number of bacterial cells to 7.0 × 10^8^ CFU/mL using PBS. To obtain heat-killed bacteria, the *C. acnes* suspension (7.0 × 10^8^ CFU/mL) was heated at 80°C for 30 min (2). The heat-killed *C. acnes* isolates were stored at 4°C and centrifuged at 5,000 rpm for 10 min before use. Heat-killed *C. acnes* reportedly induces an inflammatory response similar to viable *C. acnes* (28).

### Cell viability assay

Cell viability was evaluated using the Cell Counting Kit-8 (CCK-8, Dojindo Laboratories), following the manufacturer's instructions. SZ95 cells were seeded in 96-well plates at a density of 8 × 103 cells/well. After 24 h, the cells were treated with varying concentrations of particulate matter (PM) (0, 0.5, 1, 5, 10 μg/cm^2^) and *C. acnes* (0, 100, 200, 300, 500, 1,000 MOI [Multiplicity of Infection]) for 24 h. Then, 10 μL of CCK-8 solution was added to each well and incubated at 37°C for 3 h. Absorbance was measured at 450 nm.

### RNA isolation and qRT-PCR

SZ95 cells were seeded in six-well plates (2 × 105 cells/well). After 24 h, the medium was replaced with serum-free medium. Cells were exposed to *C. acnes* for 18 h, followed by PM exposure for 6 h. Total RNA was isolated using TRIzol reagent, and cDNA was synthesized using the RevertAid First Strand cDNA Synthesis Kit. qRT-PCR was performed using PowerUp™ SYBR^®^ Green Master Mix, and gene expression was normalized to GAPDH.

### Western blot analysis

SZ95 cells were lysed in RIPA buffer containing a protease and phosphatase inhibitor cocktail. Protein concentration was determined using a bicinchoninic acid assay, and proteins were separated by SDS-PAGE and transferred to PVDF membranes. Membranes were blocked with 5% skim milk and incubated with primary antibodies overnight, followed by HRP-conjugated secondary antibodies. Protein expression was visualized using the EZ-Western Lumi Femto Kit and normalized to GAPDH.

### Nile Red O staining

SZ95 cells were seeded in 24-well plates (2 × 104 cells/well) and treated with *C. acnes* for 18 h, followed by PM for 6 h. Cells were fixed with 4% paraformaldehyde, stained with Nile Red O dye, and counterstained with DAPI. Cells were visualized using a confocal fluorescence microscope (Leica^®^ Microsystems CMS GmbH).

### Immunofluorescence

Cells were treated similarly as in Nile Red O staining. After fixation and permeabilization, cells were blocked with 1% BSA and incubated with primary antibodies targeting SREBP-1, COX-2, p-NFκB p65, FAS, and PPARγ overnight. Secondary antibodies (Alexa Fluor 488) were applied, and cells were visualized using a confocal microscope.

### Statistical analysis

*In vitro* assays were conducted at least in triplicate. The effects of PM were compared between the treatment and control groups using one-way analysis of variance (ANOVA) and Tukey's multiple-comparison *post-hoc* test. Data were analyzed using one-way ANOVA with Bonferroni's multiple-correction test algorithm. Differences between groups were considered significant at *P* < 0.05. Statistical analyses were performed using GraphPad Prism (version 8.0; GraphPad Software, Inc., San Diego, CA, USA).

## Results

### Effects of PM and C. acnes on SZ95 cell viability

Cell viability was measured using a CCK assay kit. At a PM concentration of 10 μg/cm^2^, cell viability was ~90% compared with that of the control after 24 h of exposure (Supplementary Figure 1A). *C. acnes* did not exhibit toxicity to the cells at any tested concentration (Supplementary Figure 1B).

### Effects of PM on the mRNA expression levels of inflammatory and lipogenic genes in *C. acnes*-stimulated SZ95 cells

**Figure 1:**
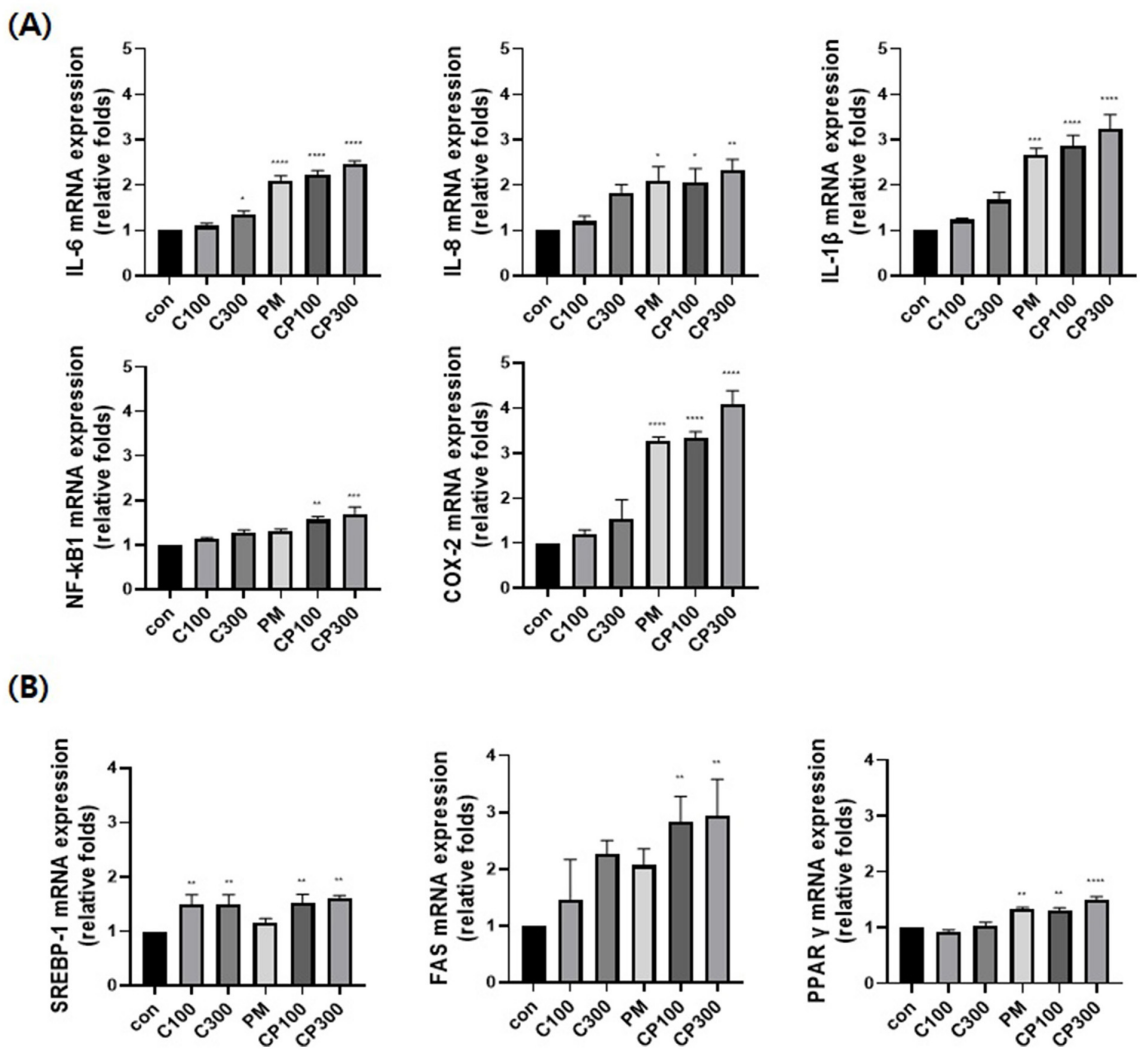
Effect of PM on the mRNA expression levels of inflammatory and lipogenic genes in *C. acnes*-treated SZ95 cells. The effect of PM (10 μg/cm^2^) on SZ95 cells treated with *C. acnes* (100, 300 MOI) for 6 h was investigated; qRT-PCR was conducted to confirm the mRNA expression levels. **(A)** The expression levels of pro-inflammatory cytokines, such as IL-6, IL-8, and IL-1β, and markers, such as NFκB1 and COX-2, were higher in the PM + *C. acnes* co-treatment group than in the control group. **(B)** Lipogenic genes, such as *SREBP-1, FAS*, and *PPAR*γ, also exhibited increased expression in the PM + *C. acnes* co-treatment group relative to those in the control group. Data are expressed as the mean + standard error of the mean. *P < 0.05, **P < 0.01, ***P < 0.001, ****P < 0.0001 vs. control. Con, control; C100, *C. acnes* 100 MOI; C300, *C. acnes* 300 MOI; CP, *C. acnes* + PM co-treatment group.

The mRNA expression levels of pro-inflammatory cytokines, such as NFκB1, IL-6, IL-8, IL-1β, and COX-2, were evaluated. The expression levels of these cytokines increased in the PM + *C. acnes* co-treatment group compared with those in the control group (Figure 1A). Additionally, the expression of lipogenesis-related transcription factors and enzymes, such as SREBP-1, FAS, and PPARγ, was confirmed. The expression levels of SREBP-1, FAS, and PPARγ were also higher in the PM + *C. acnes* co-treatment group than in the control group (Figure 1B).

### Effects of PM on the protein expression levels of inflammatory and lipogenic genes in *C. acnes*-stimulated SZ95 cells

**Figure 2:**
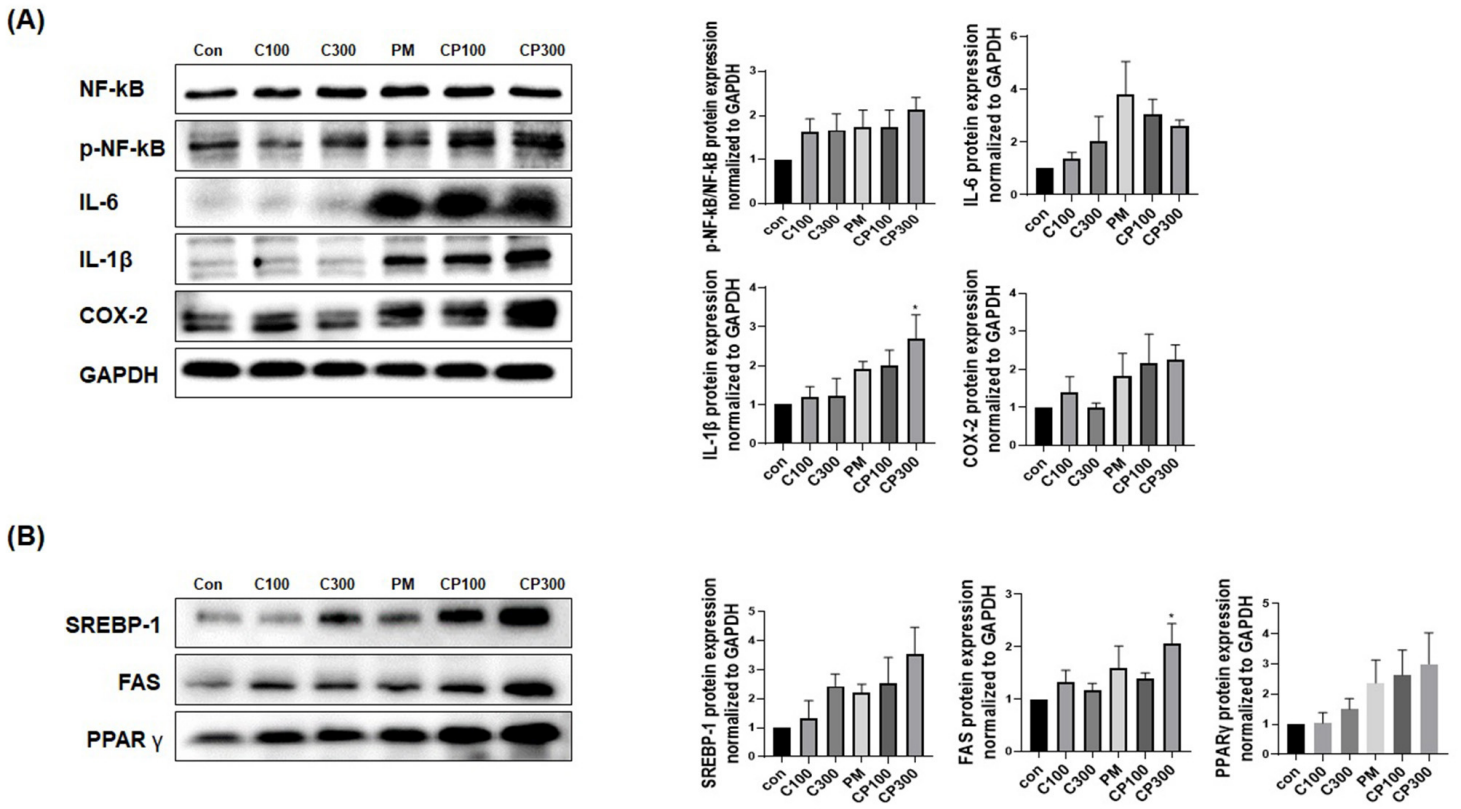
Effects of PM on the protein expression levels of inflammatory and lipogenic genes in *C. acnes*-treated SZ95 cells. The expression levels of proteins involved in inflammation and lipogenesis were investigated in SZ95 cells treated with PM (10 μg/cm^2^) and *C. acnes* (100, 300 MOI). Protein levels confirmed via Western blot analysis demonstrated that **(A)** PM induced the expression of pro-inflammatory cytokines, such as NFκB, p-NFκB, IL-6, IL-1β, and COX-2, with the PM + *C. acnes* co-treatment group displaying a more marked increase in inflammation. **(B)** PM increased the expression levels of lipogenic genes, such as *SREBP-1, FAS*, and *PPAR*γ, with the expression being significantly higher in the PM + *C. acnes* co-treatment group. Con, control; C100, *C. acnes* 100 MOI; C300, *C. acnes* 300 MOI; CP, *C. acnes* + PM co-treatment group.

The protein expression levels of pro-inflammatory cytokines, such as NFκB1, p-NFκB1, IL-6, IL-8, IL-1β, and COX-2, were found to be elevated in the *C. acnes* + PM co-treatment group (Figure 2A). Similarly, the protein expression levels of lipogenic genes, including *SREBP-1, FAS*, and *PPAR*γ, increased in the *C. acnes* + PM co-treatment group compared with those in the control group (Figure 2B). No significance was noted, except for IL-1β and *FAS*.

### Immunofluorescence staining to confirm the effects of PM and *C. acnes* on inflammatory cytokines and lipogenic genes in SZ95 cells

**Figure 3:**
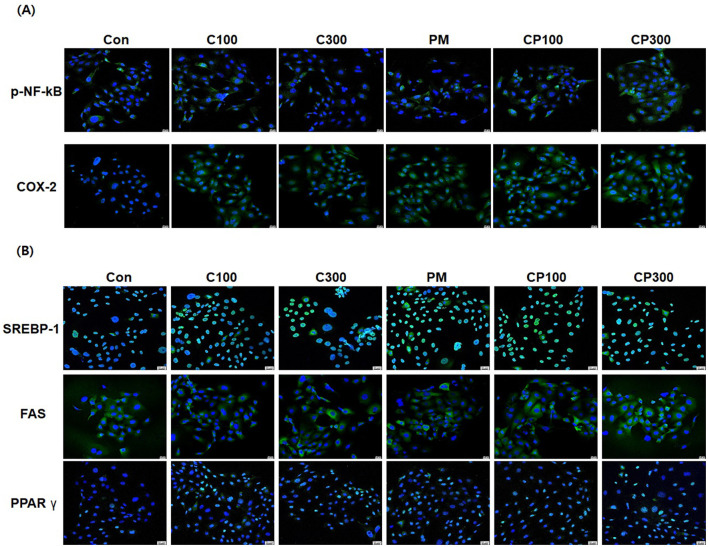
Immunofluorescence staining to confirm the effects of PM and *C. acnes* on inflammatory cytokines and lipogenic genes in SZ95 cells (×400 magnification). SZ95 cells were treated with *C. acnes* (100, 300 MOI) for 18 h, followed by treatment with PM at a concentration of 10 μg/cm^2^ for 6 h. **(A)** p-NFκB and COX-2 expression was increased by PM treatment, with a significant increase observed in the PM + *C. acnes* co-treatment group compared with that in the control group. **(B)** PM induced the nuclear translocation of SREBP-1 and PPARγ and increased the expression of FAS. Expression levels were more elevated in the PM + *C. acnes* co-treatment group (scale bar = 20 μm). Con, control; C100, *C. acnes* 100 MOI; C300, *C. acnes* 300 MOI; CP, *C. acnes* + PM co-treatment group.

The results demonstrated an elevation in the expression of pro-inflammatory cytokines, such as p-NFκB and COX-2, in the *C. acnes* + PM co-treatment group compared with that in the control group (Figure 3A). Similarly, the expression of lipogenic genes, including *SREBP-1, FAS*, and *PPAR*γ, was higher in the *C. acnes* + PM co-treatment group than in the control group (Figure 3B).

### Preventative effects of afzelin on the PM-induced expression of inflammatory and lipogenic genes in *C. acnes*-treated SZ95 sebocytes

**Figure 4:**
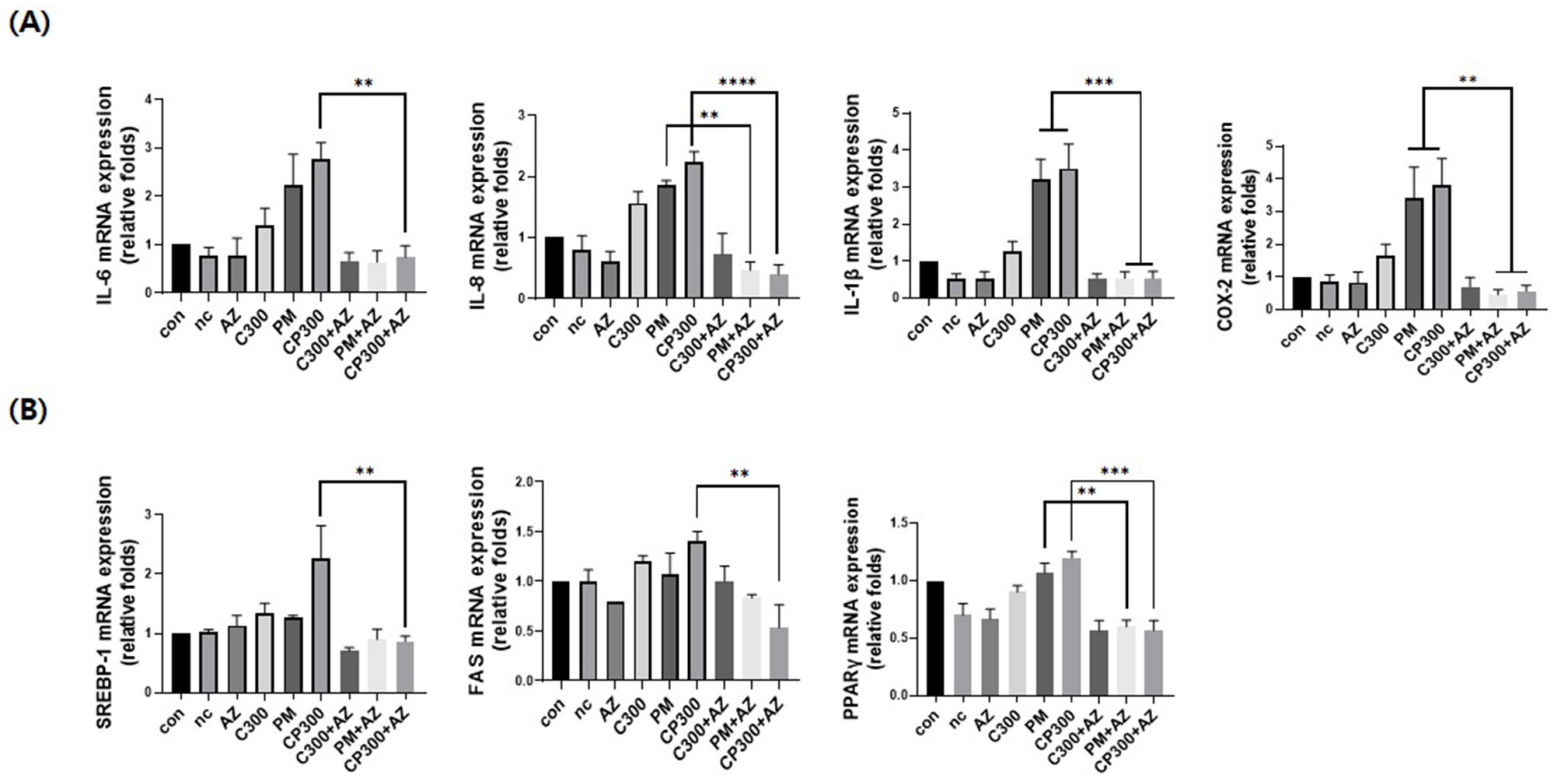
Afzelin suppressed inflammation and lipogenesis in PM-treated SZ95 sebocytes. Afzelin (500 μM) was administered to *C. acnes*-treated, PM-exposed SZ95 sebocytes for 6 h. **(A)** Relative mRNA expression levels of IL-6, IL-8, IL-1b, and COX-2 in SZ95 cells following 6-h treatment with PM (10 μg/cm^2^) and afzelin (500 μM). **(B)** Relative mRNA levels of SREBP-1, FAS, and PPARγ after 6-h treatment with afzelin. Expression was normalized to GAPDH. Data are expressed as the mean + standard error of the mean from three independent experiments. **P < 0.01, ***P < 0.001, ****P < 0.0001 vs. PM or/and CP300. Con, control; nc, negative control; C300, *C. acnes* 300 MOI; CP, *C. acnes* + PM co-treatment group; AZ, afzelin.

As shown in Figure 4A, afzelin significantly downregulated the mRNA expression levels of pro-inflammatory cytokines and enzymes, including IL-6, IL-8, IL-1β, and COX-2, in *C. acnes*-treated, PM-exposed SZ95 sebocytes. Additionally, afzelin suppressed the increased expression of lipogenic genes, including *SREBP-1, FAS, and PPAR*γ, in the afzelin-treatment group compared with that in the respective *C. acnes*- and PM-treatment groups (Figure 4B). These results suggest that afzelin suppressed inflammation and lipogenesis in *C. acnes*-treated, PM-exposed SZ95 sebocytes.

### Afzelin regulated the PM-induced protein expression of inflammatory and lipogenic genes in *C. acnes*-treated SZ95 cells

**Figure 5:**
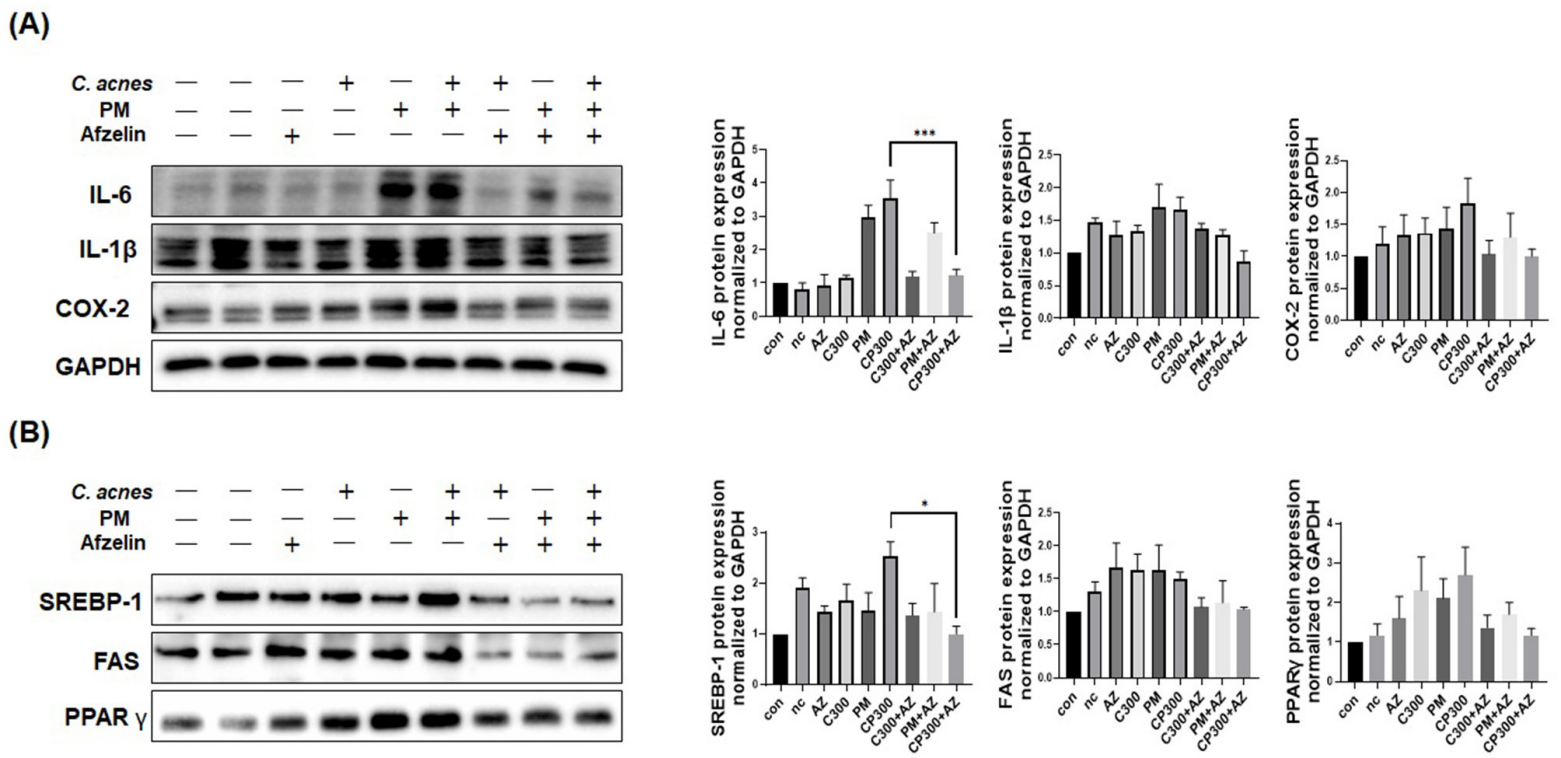
Effects of afzelin on the expression of PM-induced inflammatory and lipogenic genes in *C. acnes*-treated SZ95 sebocytes. Afzelin (500 μM) was administered to *C. acnes*-treated, PM-exposed SZ95 sebocytes for 6 h. **(A)** The protein expression levels of IL-6, IL-1b, and COX-2 in SZ95 cells following 6-h treatment with PM (10 μg/cm^2^) and afzelin (500 μM). **(B)** Protein expression levels of SREBP-1, FAS, and PPARγ after 6-h treatment with afzelin. Expression was normalized to GAPDH. Data are expressed as the mean + standard error of the mean from three independent experiments. *P < 0.05, ***P < 0.001 vs. CP 300. Con, control; nc, negative control; C300, *C. acnes* 300 MOI; CP, *C. acnes* + PM co-treatment group; AZ, afzelin.

Our results revealed that afzelin downregulated the protein expression of pro-inflammatory cytokines and enzymes, including IL-6, IL-1β, and COX-2, induced by *C. acnes* and PM (Figure 5A). Moreover, afzelin diminished the protein expression levels of lipogenic genes, such as *SREBP-1, FAS*, and *PPAR*γ, compared with the respective *C. acnes* and PM treatments (Figure 5B). No significance was observed, except for IL-6 and *SREBP-1*. Our findings demonstrate that afzelin can attenuate the inflammatory and lipogenic responses in PM-exposed SZ95 sebocytes.

### Inhibitory effect of afzelin on lipid-droplet accumulation in PM- and *C. acnes*-treated SZ95 cells

PM treatment augmented the intracellular accrual of lipid droplets, comprising neutral lipids, in the cytoplasm surrounding the nucleus. Notably, the *C. acnes* + PM co-treatment group exhibited a more pronounced increase in lipid accumulation. Nonetheless, afzelin treatment significantly mitigated PM-induced lipid-droplet accumulation in *C. acnes-*stimulated SZ95 cells (Supplementary Figure 2).

## Discussion

We investigated the effects of PM on inflammation and lipid synthesis in *C. acnes*-treated SZ95 cells. Our findings revealed that PM increases the expression of phosphorylated NF-κB and its subfactors, IL-6, IL-8, IL-1β, and COX-2. Additionally, PM upregulated the expression of lipogenesis-related genes, such as *SREBP-1, FAS*, and *PPAR*γ, and promoted the accumulation of intracellular lipid droplets in the cytoplasm. Notably, this increase in expression was significantly greater in the PM + *C. acnes* co-treatment group than in the groups exclusively treated with either PM or *C. acnes*. Based on our findings, PM can increase inflammation, lipid synthesis, and the accumulation of lipid droplets, potentially contributing to sebum composition changes (29). These results indicate that PM can aggravate acne by increasing lipid synthesis and inflammation via activation of the NF-κB pathway in *C. acnes*-treated SZ95 cells.

Acne is a multifactorial inflammatory dermatosis with numerous contributing factors. Among these, increased sebum secretion is considered a principal factor (30), and alterations in sebum composition presumably play a significant role in acne development. Although a well-established association exists between acne severity and facial sebum secretion (31, 32), recent research has indicated that sebum production may not directly precipitate acne development but may instead influence inflammatory changes in the skin (33).

PM is known to trigger the activation of the NF-κB signaling pathway, which has been implicated in the generation of inflammatory cytokines (2, 5, 34). The increased activity of NF-κB observed in clinical acne lesions underscores its critical role in the disorder's pathophysiology (35, 36). Upon activation, NF-κB migrates to the nucleus and initiates the upregulation of inflammatory genes, such as those encoding COX-2. Indeed, the inflammatory milieu in sebocytes, as evidenced by increased levels of cytokines, including IL-6, IL-8, and IL-1β, and the lipid metabolism enzyme COX-2, is reportedly elevated in acne (37).

While numerous studies have linked the effects of PM to skin diseases, research specifically addressing the role of PM in sebaceous glands, where acne manifests, is relatively scarce. To the best of our knowledge, the impact of PM on *C. acnes*-treated sebocytes has not yet been reported. Based on our findings and those of previous studies (3, 38, 39), PM potentially induces inflammation in an environment conducive to the proliferation of acne-causing bacteria, thereby exacerbating acne via enhanced inflammation and alterations in lipid composition. NF-κB-induced oxidative stress may transform the pilosebaceous unit into a breeding ground for anaerobic bacteria, creating conditions favorable for the survival of acne-causing bacteria. Additionally, this potentially leads to lipid peroxidation, which may exacerbate acne severity (40).

In addition, our study investigated the preventative effects of afzelin on PM-induced inflammation and lipogenesis in C. acnes-treated SZ95 sebocytes. The results indicate that afzelin significantly downregulates the expression of inflammation- and lipogenesis-related genes and proteins. However, the reduction in protein expression was statistically significant only for IL-6 and SREBP-1. The lack of statistical significance in IL-6 and SREBP-1 protein levels may stem from variability in cellular responses to PM and *C. acnes* or suboptimal experimental conditions, such as timing and stimulus concentrations. Additionally, post-translational modifications or compensatory mechanisms may attenuate changes at the protein level despite significant mRNA expression. It is also possible that afzelin's effects are more pronounced on other mediators. Further studies with larger sample sizes and optimized conditions are needed to clarify these findings.

Moreover, this study examined the effect of afzelin on lipid-droplet accumulation in SZ95 cells. PM treatment led to an increase in lipid droplets within the cytoplasm, especially around the nucleus. This accumulation was more pronounced in the *C. acnes* + PM co-treatment group. Notably, afzelin significantly reduced PM-induced lipid-droplet accumulation in these cells. These findings collectively suggest that afzelin exerts a protective effect against PM-induced inflammation and lipogenesis in *C. acnes*-treated sebocytes.

Taken together, our study confirms that PM incites inflammation and escalates the expression of lipogenic genes in *C. acnes*-treated SZ95 cells, while afzelin holds promise as a therapeutic agent for environmental pollutant-exacerbated conditions, such as acne vulgaris, by mitigating both inflammatory responses and lipid synthesis. The findings of this study underscore the need to consider the size of particulate matter (PM) when evaluating its impact on skin conditions like acne. Smaller particles, such as PM2.5, are capable of deeper skin penetration and carry more toxic substances, which may amplify oxidative stress and inflammatory responses. This mechanism could explain why PM exposure exacerbates acne symptoms. Larger particles, while less capable of deep penetration, may still provoke localized irritation and inflammation. Understanding these size-dependent effects is essential for designing effective preventative and therapeutic interventions tailored to mitigate the dermatological impact of PM exposure. Future studies focusing on specific PM sizes will help further elucidate these mechanisms.

Notwithstanding, the interpretation of our results is subject to certain limitations. First, the upstream mechanisms of NF-κB, including oxidative stress and aryl hydrocarbon receptor expression, were not explored. Second, further investigations are warranted to elucidate specific changes in sebum composition. In acne pathogenesis, not only the increase in sebum production but also the alterations and oxidation of sebum composition are significant. For instance, the variation in lipid content, particularly squalene oxidation in individuals with acne, has been implicated in the development of lesions, bacterial toxicity, and inflammatory processes (41, 42). Third, our study was performed *in vitro*. To ascertain the biological significance of these findings, additional studies employing animal models and clinical trials are essential. To decode the mechanism behind our results, identifying specific biomarkers directly associated with acne and examining the link between lipid synthesis pathways and inflammatory responses are imperative. Further studies are warranted to elucidate the underlying mechanisms and explore the clinical relevance of these findings.

The insights from our study may be instrumental in deciphering the relationship between air pollution and acne development, including its underlying mechanisms. Modulating the expression of inflammatory markers, cytokines, and lipogenic genes may help identify potential therapeutic targets for mitigating the PM-induced exacerbation of acne in the future.

## Data Availability

The datasets presented in this article are not readily available because some datasets may prohibit redistribution, requiring users to direct others to the original source rather than sharing the dataset themselves. Requests to access the datasets should be directed to kyky@cauhs.or.kr.

## Supplementary material

Supplementary File 1 Primer sequences for qRT-PCR used in this study.

Supplementary Figure 1 Effects of PM and *C. acnes* on SZ95 cell viability. The cytotoxicity of PM and heat-killed *C. acnes* to SZ95 cells was measured using the CCK-8 assay. **(A)** SZ95 cells were treated with various PM concentrations (0, 0.5, 1, 5, and 10 μg/cm^2^) for 6–24 h. **(B)** SZ95 cells were treated with various heat-killed *C. acnes* concentrations (100, 200, 300, 500, and 1,000 MOI) for 6–24 h. Data are expressed as the mean + standard error of the mean. ^**^*P* < 0.01. Conc, concentration.

Supplemental Figure 2 Effect of afzelin on PM-induced lipid-droplet increase in *C. acnes*-treated SZ95 sebocytes (×400 magnification). SZ95 cells were treated with *C. acnes* (300 MOI) for 18 h, followed by PM (10 μg/cm^2^) treatment for 6 h. Nile Red O staining and subsequent fluorescence microscopy were used to observe lipid-droplet changes in SZ95 cells. PM and *C. acnes* increased the number of lipid droplets in SZ95 cells, and this increase was more pronounced in the PM + *C. acnes* co-treatment group. Notably, afzelin treatment significantly inhibited the PM-induced formation of lipid droplets in *C. acnes*-treated SZ95 cells (scale bar = 20 μm). Con, control; C300, *C. acnes* 300 MOI; CP, *C. acnes* + PM co-treatment group; AZ, afzelin.
