# Supplementary material for: Effect of afzelin on inflammation and lipogenesis in particulate matter-stimulated C. acnes-treated SZ95 sebocytes
Source: Front Med (Lausanne). 2025 Jan 29;12:1518382. doi: 10.3389/fmed.2025.1518382 (PMC11813740; doi:10.3389/fmed.2025.1518382)
Supplement: Supplementary File 1 — Primer sequences for qRT-PCR used in this study. [file Table_1.docx]

**<Afzelin result primer>**

**GAPDH(F):** GAT CAT CAG CAA TGC CTC CT

**GAPDH(R):** TGT GGT CAT GAG TCC TTC CA

**SREBP-1(F):** GCCATGGATTGCACTTT

**SREBP-1(R):** CAAGAGAGGAGCTCAATG

**FAS(F):** TGT GGA CAT GGT CAC GGA C

**FAS(R):** GGC ATC AAA CCT AGA CAG GTC

**PPAR gamma(F):** TAC TGT CGG TTT CAG AAA TGC C

**PPAR gamma(R):** GTC AGC GGA CTC TGG ATT CAG

**IL-1B(F):** ACG ATG CAC CTG TAC GAT CA

**IL-1B(R):** GGA GGT GGA GAG CTT TCA GT

**IL-6(F):** TAC ATC CTC GAC GGC ATC TC

**IL-6(R):** AGT GCC TCT TTG CTG CTT TC

**CXCL8(F):** GTT CCA CTG TGC CTT GGT TT

**CXCL8(R):** GCT TCC ACA TGT CCT CAC AA

**COX-2(F):** GCA ATA ACG TGA AGG GCT GT

**COX-2(R):** CGG GAA GAA CTT GCA TTG AT
