# Supplementary figures and images for: Effect of afzelin on inflammation and lipogenesis in particulate matter-stimulated C. acnes-treated SZ95 sebocytes
Source: Front Med (Lausanne). 2025 Jan 29;12:1518382. doi: 10.3389/fmed.2025.1518382 (PMC11813740; doi:10.3389/fmed.2025.1518382)

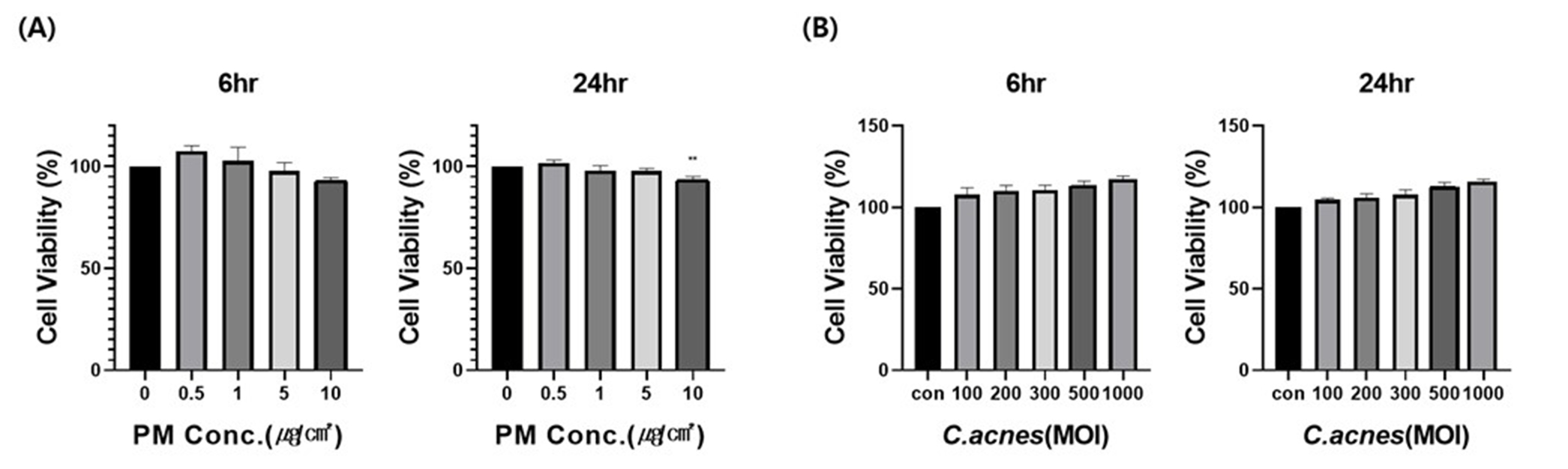

Supplement: Supplementary Figure 1 — Effects of PM and C. acnes on SZ95 cell viability. The cytotoxicity of PM and heat-killed C. acnes to SZ95 cells was measured using the CCK-8 assay. (A) SZ95 cells were treated with various PM concentrations (0, 0.5, 1, 5, and 10 μg/cm2) for 6–24 h. (B) SZ95 cells were treated with various heat-killed C. acnes concentrations (100, 200, 300, 500, and 1,000 MOI) for 6–24 h. Data are expressed as the mean + standard error of the mean. **P < 0.01. Conc, concentration. [file Image_1.jpeg]

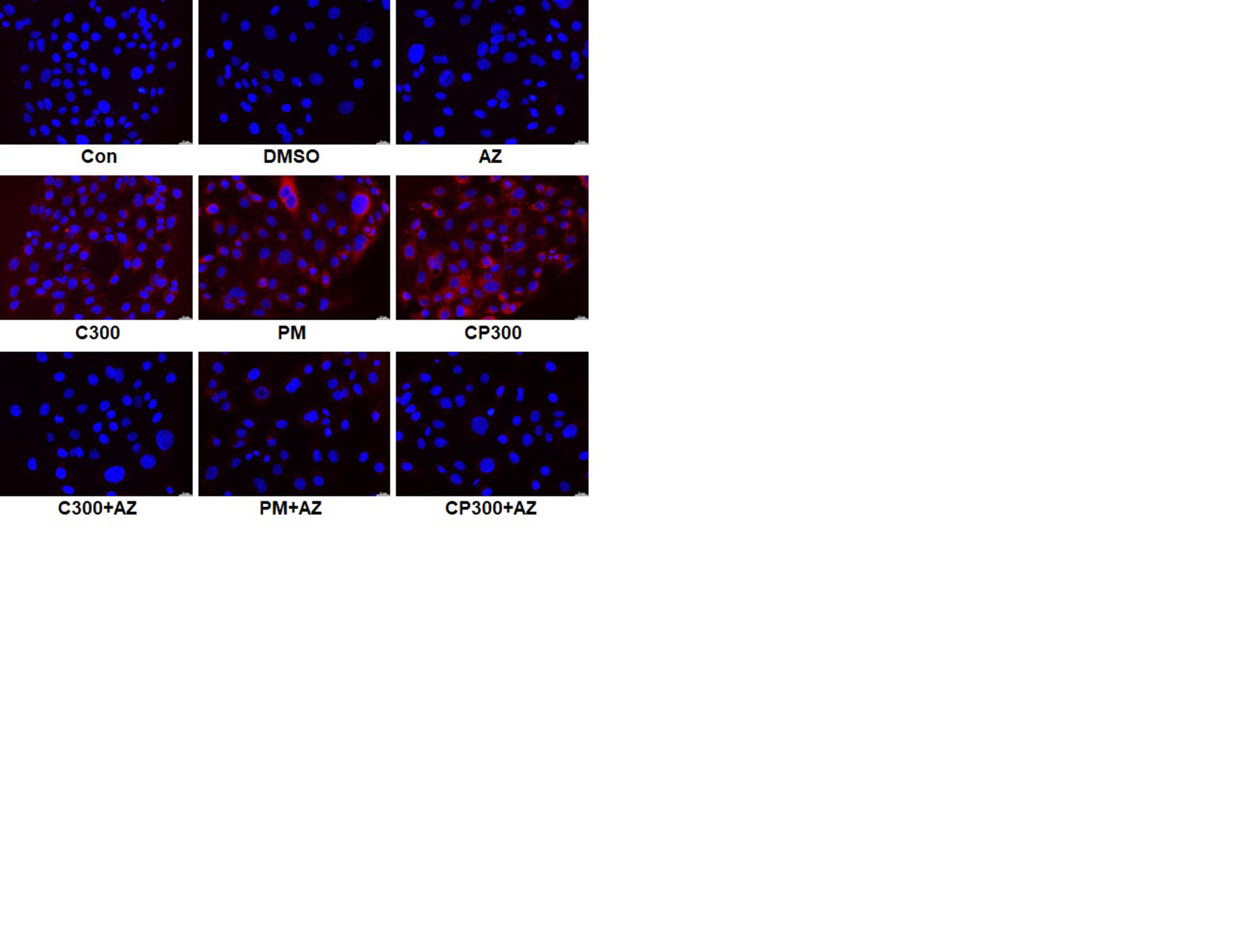

Supplement: Supplemental Figure 2 — Effect of afzelin on PM-induced lipid-droplet increase in C. acnes-treated SZ95 sebocytes (×400 magnification). SZ95 cells were treated with C. acnes (300 MOI) for 18 h, followed by PM (10 μg/cm2) treatment for 6 h. Nile Red O staining and subsequent fluorescence microscopy were used to observe lipid-droplet changes in SZ95 cells. PM and C. acnes increased the number of lipid droplets in SZ95 cells, and this increase was more pronounced in the PM + C. acnes co-treatment group. Notably, afzelin treatment significantly inhibited the PM-induced formation of lipid droplets in C. acnes-treated SZ95 cells (scale bar = 20 μm). Con, control; C300, C. acnes 300 MOI; CP, C. acnes + PM co-treatment group; AZ, afzelin. [file Image_2.jpeg]
